# Diversity of Microscopic Fungi

**DOI:** 10.3390/jof12020108

**Published:** 2026-02-04

**Authors:** Miloš Stupar

**Affiliations:** Faculty of Biology, University of Belgrade, Studentski Trg 16, 11000 Belgrade, Serbia; smilos@bio.bg.ac.rs

## 1. Introduction

Microscopic fungi constitute one of the most varied and ecologically significant groups of organisms on Earth; nonetheless, a substantial portion of their richness remains uncharted and little studied. Microfungi are ubiquitous and ecologically versatile organisms that inhabit almost all environments, from soils and plant tissues to aquatic, built, and extreme habitats such as caves, deserts, and polar regions. Yeasts, molds, and microcolonial fungi are “essential players” in both natural and human-made environments. They play key ecological roles as decomposers driving nutrient cycling, as mutualists forming symbioses with plants and animals, and as parasites or pathogens influencing population dynamics. Through these functions, microfungi strongly shape ecosystem processes, stability, and resilience. Thus, scientific studies regarding these microorganisms are crucial in improving ecosystem sustainability, public health, cultural heritage preservation, and innovation in biotechnology [1,2,3].

Recent advances in molecular techniques, particularly high-throughput sequencing and metagenomic approaches, have fundamentally transformed the study of microfungal diversity. Culture-independent methods now enable the detection and accurate taxonomic characterization of fungal taxa directly from environmental samples, including rare, cryptic, and unculturable species that were previously inaccessible using classical cultivation-based approaches. These techniques have greatly improved our ability to identify fungal community structure, diversity, and functional potential across a wide range of ecosystems. As sequencing technologies and bioinformatic tools continue to develop, metagenomic approaches are expected to further expand our understanding of microfungal diversity and its broader ecological roles, as well as practical implications in fields such as biotechnology, agriculture, environmental monitoring, and human and plant health [4,5,6,7].

## 2. Overview of Published Articles

This Special Issue comprises 10 original research articles and one review paper that are focused on various aspects of biology of microfungi, providing insights from several ecological viewpoints. Research groups from Austria (Derksen et al. (contribution 1)), China (Ding et al. (contribution 2), Xiong et al. (contribution 6), Pu et al. (contribution 8), Guan et al. (contribution 9) and Han et al. (contribution 10)), Mexico (Soto-Plancarte et al. (contribution 4)), Portugal (Pereira et al. (contribution 7)), Serbia (Savković et al. (contribution 11)), Taiwan (Derksen et al. (contribution 1)), Thailand (Wonglom et al. (contribution 3)), the UK (Derksen et al. (contribution 1)), and the USA (Soto-Plancarte et al. (contribution 4), Joseph et al. (contribution 5), Xiong et al. (contribution 6), Pu et al. (contribution 8) and Guan et al. (contribution 9)) have contributed novel findings to this Special Issue. The topics covered include biodeteriogenic microfungi from museum depots and archives (Derksen et al. (contribution 1)), indoor air mycobiota (Derksen et al. (contribution 1)), soil fungi (Ding et al. (contribution 2)), plant pathogenic fungi (Wonglom et al. (contribution 3), Soto-Plancarte et al. (contribution 4), Pereira et al. (contribution 7) and Han et al. (contribution 10)), phyllosphere colonizers (Guan et al. (contribution 9)), entomopathogenic fungi (Xiong et al. (contribution 6) and Pu et al. (contribution 8)), symbiont colonization of Ambrosia beetles (Joseph et al. (contribution 5)), and fungal cave dwellers (Savković et al. (contribution 11)). Ding et al. (contribution 2), Guan et al. (contribution 9) and Han et al. (contribution 10) provide descriptions of novel fungal species and as such make significant contributions to cataloguing Earth’s biological diversity.

Derksen et al. (contribution 1) assessed fungal risks in nine museum depots and archives by combining two years of indoor microclimate monitoring with culture-based and metagenomic analyses of fungal abundance and diversity. The dominant fungal genera differed between methodological approaches, but patterns across sites were primarily driven by geographic location, surrounding environments, and indoor microclimatic variability. Their findings highlight biological degradation as a growing threat linked to climate change and suggest the need to rethink mold prevention strategies beyond strict climate control alone.

Ding et al. (contribution 2) describe seven new *Cunninghamella* species from China, identified using an integrated taxonomic framework combining morphology, multilocus phylogenetics, and physiological traits. Phylogenetic analyses placed the new taxa within a well-supported clade alongside *C. bainieri* and *C. verticillata*, with each species distinguished by clear morphological or geographic features. Their findings expand the genus *Cunninghamella* to 63 species and contribute to a broader understanding of early-diverging fungal diversity and the evolution of Mucoromycota.

Furthermore, Wonglom et al. (contribution 3) investigated a newly observed leaf spot disease on peach palm (*Bactris gasipaes*) seedlings in southern Thailand and identified the causal agents through pathogenicity tests and morphological and molecular analyses. The disease was attributed to *Colletotrichum fructicola*, *C. theobromicola*, and *Fusarium pernambucanum*, representing the first report of these fungi affecting peach palm in Thailand. Fungicide assays demonstrated differing sensitivities among the pathogens, providing practical information for developing effective disease management strategies. Similarly, Soto-Plancarte et al. (contribution 4) surveyed *Phytophthora* species in ornamental plant nurseries across Mexico, addressing a poorly studied but economically important phytosanitary problem. Seven *Phytophthora* species were identified using morphological traits and molecular analyses, including the first record of *P. kelmanii* in Mexico, as well as potentially undescribed taxa and a putative interspecific hybrid. Their findings also document nursery plants as vectors of *Phytophthora* to home gardens and identify *Cestrum nocturnum* and *Solanum ovigerum* as new hosts worldwide.

On the other hand, Joseph et al. (contribution 5) examined specificity and dynamics of fungal symbiont colonization in ambrosia beetle mycangia, focusing on interactions involving the invasive pathogen *Harringtonia lauricola*. The results showed strong host fungus compatibility in *Xyleborus affinis* but limited colonization in a related beetle species, with mycangial occupancy influenced by fungal nutritional status and exhibiting rapid, dynamic partner turnover. A new image cytometry-based method was developed to directly quantify mycangial contents, providing novel insights into ambrosia beetle–fungus associations and their ecological variability.

Xiong et al. (contribution 6) report the first mitochondrial genomes for the insect-pathogenic fungal genus *Moelleriella*, based on seven species with mitogenome sizes ranging from ~41 to 96 kb. Comparative and evolutionary analyses showed conserved gene order and purifying selection across core genes, while extensive intron variation was identified as the main driver of genome size differences. Phylogenetic results place *Moelleriella* in a well-supported clade closely related to *Metarhizium*, providing new insights into the taxonomy and evolutionary genomics of the genus.

Furthermore, Pereira et al. (contribution 7) provide a first assessment of phytopathogenic fungal diversity associated with foliar lesions on ornamental palms in temperate Portugal. Using morphological and molecular approaches, a highly diverse fungal community was revealed, dominated by a few common genera but containing numerous rare taxa, with a community structure shaped by climate, host species, and fungal co-occurrence patterns. The results demonstrate that temperate ornamental palms harbor fungal assemblages distinct from tropical palms, highlighting foliar lesions as hyperdiverse microhabitats with important implications for disease risk under changing climatic conditions.

Pu et al. (contribution 8) reconstructed the evolutionary history and biogeography of the insect-pathogenic fungal family Cordycipitaceae using multilocus molecular clock analyses, indicating a Paleogene origin of *Akanthomyces sensu lato* in Asia with most diversification occurring during the Neogene. Integrating phylogenetic and morphological data, the authors describe two new species and report several new host and regional records across multiple genera within the family. Overall, the findings refine Cordycipitaceae phylogeny and substantially expand knowledge of its diversity, ecology, and evolutionary development.

Guan et al. (contribution 9) investigated phyllosphere fungi of the order Diaporthales from diseased leaves of three tree species in Fujian Province, China. With the use of detailed morphological observations and multilocus phylogenetic analyses, three new species, *Diaporthe wuyishanensis*, *Gnomoniopsis wuyishanensis*, and *Paratubakia schimae*, were identified and described. These findings enhance our knowledge of the diversity of diaporthalean fungi associated with Asian tree phyllospheres and highlight the presence of previously uncharacterized taxa.

Han et al. (contribution 10) investigated Nectriaceae fungi causing leaf spots on *Cinnamomum camphora* trees in southern China. Molecular and morphological analyses of 54 isolates identified two as *Calonectria crousiana* and 52 as a newly described genus and species, *Recticladiella inexpectata*, distinguished by unique conidiophore morphology and DNA sequence differences. Pathogenicity tests confirmed that both *R. inexpectata* and *C. crousiana* cause leaf lesions on *Ci. camphora*, indicating their role as pathogens with a broad distribution in the region.

Finally, Savković et al. (contribution 11) analyzed NCBI sequence data to uncover global patterns in fungal diversity in cave environments, identifying 445 species across 394 genera from 1447 sequences. Ascomycota, particularly the class Eurotiomycetes, dominated, with *Penicillium* and *Aspergillus* showing the highest species richness, while *Pseudogymnoascus destructans* was the most frequently recorded species. Caves’ stable, nutrient-limited microhabitats support rare and cryptic fungi, and advanced molecular methods are increasingly revealing previously undiscovered cave fungal diversity.

## 3. Outlook and Prospects

Most microfungal diversity remains undescribed, especially in understudied and extreme environments such as caves, deep soils, aquatic systems, and built environments. Integrative taxonomy combining culturing, genomics, and environmental sequencing will continue to uncover novel taxa and evolutionary lineages [8,9,10]. Further studies on microscopic fungi will improve our understanding of nutrient cycling, ecosystem resilience, and microbial interactions under environmental change. Moreover, microfungi are a major source of enzymes, antibiotics, bioactive compounds, and industrial metabolites. Examining unexplored lineages increases the likelihood of discovering novel compounds for pharmaceutical, agricultural, and environmental applications [11,12]. To conclude, future research into microscopic fungi will move toward integrative, multi-omics, and ecology-driven approaches, positioning microfungal diversity studies as central in both fundamental biology and practical solutions to environmental and societal challenges.
